# Anti-Müllerian Hormone as a Sensitive Marker of Ovarian Function in Young Cancer Survivors

**DOI:** 10.1155/2013/125080

**Published:** 2013-12-15

**Authors:** Maryna Krawczuk-Rybak, Elzbieta Leszczynska, Marta Poznanska, Beata Zelazowska-Rutkowska, Jolanta Wysocka

**Affiliations:** ^1^Department of Pediatric Oncology and Hematology, Medical University of Bialystok, Waszyngtona 17, 15-274 Bialystok, Poland; ^2^Department of Pediatric Laboratory Diagnostics, Medical University of Bialystok, Poland

## Abstract

We evaluated ovarian function by measuring the levels of anti-Müllerian hormone (AMH), estradiol, and gonadotropins in 83 young women treated for cancer during childhood and adolescence, and classified according to post-treatment gonadal toxicity versus 38 healthy females. *Results*. The mean AMH values were lower in the entire cohort independently of the risk group as compared to the control, whereas FSH was elevated only in the high risk group. The lowest AMH values were noted in patients after bone marrow transplantation (BMT) and those treated for Hodgkin lymphoma (HL). Nineteen patients (22.9%) had elevated FSH. They all had low AMH values. Lowered AMH values (but with normal FSH and LH) were observed in 43 patients (51.8%). There was no effect of age at the time of treatment (before puberty, during or after puberty) on AMH levels. *Conclusion*. Our results show the utility of AMH measurement as a sensitive marker of a reduced ovarian reserve in young cancer survivors. Patients after BMT and patients treated for HL, independently of age at treatment (prepuberty or puberty), are at the highest risk of gonadal damage and early menopause.

## 1. Introduction 

The use of combined chemo- and radiotherapy for childhood cancer treatment has led to an increased survival rate and posed new challenges concerning health problems, organ damage, and quality of life after anticancer therapy. The function of different tissues and organs may be differently impaired by aggressive therapy. Gonads are particularly exposed to the deteriorating effects of certain chemotherapeutics and radiotherapy; on the other hand, when survivors reach adulthood, they wish to have their own biological children [1–3]. Fertility after anticancer therapy is a very important problem known by oncologists and endocrinologists as well as by cancer survivors themselves.

In women, gonadotoxic therapy damages the primordial follicles in the ovaries, which can lead to premature menopause. Very aggressive therapy such as myeloablative therapy prior to bone marrow transplantation (BMT) or surgery/ovariectomy can lead to total sterility, whereas indirect irradiation of the ovaries and chemotherapy can result in a lowered ovarian reserve [4–6].

In the last years, the measurement of anti-Müllerian hormone (AMH) has been used as an informative marker of the ovarian reserve. AMH is a product of granulosa cells of preantral and early antral follicles, capable of growing. In healthy women, AMH measurement is useful for the determination of the reproductive life span and the time of future menopause [7, 8].

The aim of our study was to determine the ovarian reserve in young women after anticancer treatment during childhood and adolescence, using the protocols with different degrees of gonadotoxicity.

## 2. Patients and Methods

We recruited 83 young women, cancer survivors, from the Department of Pediatric Oncology and Hematology (outpatient clinic), Medical University of Bialystok. At diagnosis, they were from 0.9 to 17.8 years old (*x* = 10.5 ± 5.21), and, at examination, they were 18.78 ± 4.98 years old. They had been treated for Hodgkin lymphoma, HL (*n* = 22), Wilms tumor (*n* = 11), soft tissue sarcoma, STS (*n* = 7), neuroblastoma (*n* = 2) germinal tumors (*n* = 7), acute myeloblastic leukemia (*n* = 4), acute lymphoblastic leukemia (*n* = 22) chronic myeloblastic leukemia (*n* = 2), and non-Hodgkin lymphoma (*n* = 6). They were all treated according to international protocols; 20 received irradiation for the infradiaphragmatic area (10 with HL), irradiation for the central nervous system (CNS)—9 patients with leukemia, 6 received bone marrow transplantation (1—total body irradiation, TBI—12 Gy).

All patients were classified according to a possible degree of gonadotoxicity proposed by Wallace et al.; the risk of disturbed fertility or infertility depends on diagnosis, stage, and type of treatment (alkylating agents, radiotherapy to the pelvis/ovaries). The probability of infertility in the low risk group is less than 20% and in the medium risk group between 20 and 80%, whereas, in the high risk group, is greater than 80%.

At diagnosis, forty-five patients were in Tanner stage T1-2, *n* = 5 in T3, and *n* = 33 in T4-5. On examination, one female presented with primary amenorrhea, 65 had normal, regular menses, and 17 had irregular menses or oligomenorrhea (11 were classified as high risk group, 2 as low risk, and 4 as middle risk group). Six patients have their biological children (five treated for HL, one treated for non-Hodgkin lymphoma, NHL).

Details concerning diagnosis, age at the time of therapy, type of therapy, the interval between the end of therapy, and measurements of hormone levels are presented in Table 1, taking into consideration the risk groups proposed by Brougham and Wallace [9].

Control group was composed of 38 healthy females aged 20.68 ± 4.34.

## 3. Hormone Level Measurements

Serum concentrations of FSH, LH, and E2 were measured in the same laboratory using the commercially available immunoenzymatic kits; serum AMH levels were determined with the EIA AMH/MIS kit (Immunotech, Beckman Coulter Company/Marseille, France). All hormonal measurements were performed in the early follicular phase (2–4 days of menstrual cycle) and stored at −80°C.

The study was approved by the local Medical Ethics Committee. Hormone measurements were made after an informed consent was obtained from the patients.

## 4. Results

We found higher FSH and lower AMH levels in the entire group as compared to the control group (*P* = 0.001; *P* = 0.001, resp.), whereas the mean levels of estradiol and LH were normal. When the study group was subdivided according to the risk of gonadotoxicity, the levels of FSH were elevated only in the high risk group (18.11 ± 28.7 mIU/mL versus 5.36 ± 1.89 mIU/mL, *P* = 0.005), whereas, in the middle and low risk groups, they were comparable with the control group. AMH values were lower than those in the control group in all the three risk groups (HR group 14.14 ± 13.26 pmol/L (*P* = 0.001); MR group 14.82 ± 16.2 pmol/L (*P* = 0.019); LR group 19.44 ± 13.96 pmol/L (*P* = 0.053)). Mean serum LH and estradiol values did not differ between the risk groups and control (see Table 2).

The HR group was analyzed separately: patients diagnosed with HL irradiated and nonirradiated for the infradiaphragmatic region, patients treated for solid tumors with radiation to the infradiaphragmatic area, and patients after bone marrow transplantation. In these subgroups, AMH values were lower than those in the control group, being the lowest in patients after bone marrow transplantation (3.37 ± 2.32 pmol/mL). FSH levels were the highest in females after BMT (42.55 ± 26.55 mIU/mL) and elevated in females treated for HL with inverted Y irradiation. The values of LH and estradiol did not differ between the HR group and control (except the patients after BMT) (see Table 3).

There were 19 females (22.9%) in the study group with elevated FSH levels (>10 mIU/mL), AMH lower than 12.5 pmol/L, and normal LH levels; 12/19 derived from the HR group. They all presented low AMH values. Lowered AMH levels (yet with normal FSH and LH) were observed in 43 patients (51.8%).

We found no influence of age at the time of treatment (before puberty, during or after puberty), although AMH was lower in patients treated after puberty (13.04 ± 12.06 pmol/L) than during puberty (15.43 ± 13.65 pmol/L) and before puberty (18.52 ± 14.93 pmol/L).

## 5. Discussion

Combined anticancer treatment has improved the prognosis for young patients and at the same time has enabled us to recognize different late effects of the treatment. Diminished fertility or infertility and early menopause are the major side effects lowering life quality among cancer survivors. According to Childhood Cancer Survivor Study (CCSS), premature menopause occurs in 8% survivors and depends on age, dose of irradiation to the ovaries, and cumulative dose of alkylating agents [10]. Brougham and Wallace classified the most common cancers treated during childhood and adolescence according to the risk of subfertility resulting from gonadotoxicity. The high risk group includes patients after TBI, megachemotherapy, tumors located in pelvis and irradiated, metastatic soft tissue sarcomas, and Hodgkin lymphomas treated with alkylating agents. The risk of impaired fertility in this group is more than 80%, as compared to less than 20% in the low risk group [9]. Our knowledge concerning the toxicity of anticancer treatment enlarges, and treatment protocols change, not only for better outcome and improved survival but also for a reduction in side effects. We categorized our patients according to the type of malignancy and treatment, mainly the area of radiotherapy and total dose of alkylating agents.

To evaluate ovarian function we analyzed the levels of FSH, LH, estradiol, and AMH. In males, it is easier to evaluate gonadal function by analyzing spermiograms and hormone measurements; in females, assessment of oocyte depletion and premature ovarian failure is more difficult. AMH seems to be very useful to determine the ovarian reserve, better than the evaluation of the antral follicle count by vaginal ultrasonography or the measurement of FSH and inhibin B. The AMH level is relatively constant from mid-childhood to early adulthood, without fluctuations between pubertal stages [7, 11–13]. Elevated FSH levels were observed only in the HR group, whereas lower AMH (compared to the control group) was found in the total cohort independently of risk group. More than half (51.8%) of the patients had low AMH levels, whereas 22.9% presented with elevated FSH values. Abnormalities were most pronounced in patients after BMT, who had very low AMH values and elevated FSH. Total body irradiation and/or high doses of alkylating agents, such as cyclophosphamide or busulphane, led to ovary dysfunction. Similar results have been reported by Miyoshi et al., who found low AMH levels in 53% and high FSH in 30% of childhood cancer survivors [14]. Fong et al. observed accelerated loss of primordial follicles in females after TBI before stem cell transplantation [15]. The treatment for HL also leads to high risk of premature menopause [16, 17]. De Bruin et al. found a 12-fold higher risk of early (before the age of 40) menopause in HL survivors treated with procarbazine as an element of chemotherapy as compared to those irradiated for the supradiaphragmatic areas or paraaortic nodes [18]. Like in our study, van Beek et al. found that hormone levels were not influenced by age at treatment (before versusduring puberty) [19]. Different results have been presented for women treated at an age older than 30 years since AMH levels fall gradually due to a reduced oocyte pool [20, 21]. In the patients treated for HL, lower AMH values were noted for the irradiated and nonirradiated infradiaphragmatic areas, whereas elevated FSH levels were observed only in irradiated females. Those who are irradiated for the infradiaphragmatic region received 3 or 4 cycles with procarbazine (MOPP), and those who are nonirradiated received only two cycles. In the former, the gonadotoxic effect might result from the combined effect of chemo- and radiotherapy. Patients treated for solid tumors, irradiated for the abdomen, also presented with lower AMH levels; in that group, only one female was irradiated (44 Gy) for the pelvic area—she had primary amenorrhea and received hormonal therapy. Irradiation directly for the ovaries, especially in the total dose >15 Gy, seems to be the most important factor deteriorating gonadal function [22], although according to Wallace et al. the LD50 for human oocyte is <2 Gy [23].

We observed lowered AMH levels not only in the HR group but also in the MR or LR group, which indicates that all types of anticancer treatment affected gonadal function, even when low doses of chemotherapeutics were used. Patients treated for acute lymphoblastic leukemia, classified to the low risk group, show subtle ovarian disorders (lower estradiol levels) [24] and some of them are infertile after anticancer treatment [25]. In a prospective study performed during and after cytotoxic treatment, Brougham et al. observed progressive lowering of AMH during treatment and recovery in the low and middle risk groups between 2 and 12 months following therapy completion, thus indicating possible restoration of the pool of growing oocytes. This recovery was not observed in the high risk group, suggesting a profound loss of the primordial follicle pool [26].

Sixty-five out of 83 survivors had normal regular menstrual cycle, one had primary amenorrhea, and 17 had irregular menses or oligomenorrhea; the latter group included patients after BMT after HL treatment (HR group). The group with normal menstrual cycles contained patients with lower AMH levels and with a diminished ovarian reserve.

We found elevated FSH in the early follicular phase with normal LH and estradiol levels; this situation is characteristic of premature ovarian failure and can appear even 20 years prior to menopause [19, 27, 28]. Taken together, lowered mean AMH values in the entire cohort and a monotropic rise in FSH indicate the possibility of premature menopause [29]. Our results suggest older biological ovarian age as compared to the chronological one. Six patients had their biological children; five were treated for HL and were classified to the HR group. The peak incidence for HL is observed in older adolescents, most often over 15 years of age. Female cancer survivors should be informed that their “fertility window” is shorter than that of the general population [30].

In conclusion, our results show the utility of AMH measurement as an early, sensitive marker of a reduced ovarian reserve in young cancer survivors. Patients after conditioning therapy prior to BMT as well as patients treated for HL, independently of age at treatment (prepuberty or puberty), are at the highest risk of gonadal damage and early menopause.

## Figures and Tables

**Table 1:** Characteristic of patients classified according to different risk groups and related treatment.

Diagnosis	*n*	Age at diagnosis (y)	Age at exam (y)	Time off treatment (y)	Chemotherapy (doses of gonadotoxic chemotherapeutics)	Radiotherapy
HR	38					
HL	22	15.2 ± 2.6	21.35 ± 4.4	6.03 ± 3.8		
IIA, IIB	12				3 × MVPP + 3 × B-DOPA Dacarbazine 900 mg/m^2^ Procarbazine 3000 mg/m^2^ Nitrogen mustard 36 mg/m^2^	Supradiaphragm 20 Gy
IIIA	7				3 × MVPP + 3 × B-DOPA	Supradiaphragm 20 Gy Infradiaphragm 15 Gy
IIIB	3				4 × MVPP + 4 × B-DOPA Dacarbazine 1200 mg/m^2^ Procarbazine 4000 mg/m^2^ Nitrogen mustard 48 mg/m^2^	Supradiaphragm 20 Gy Infradiaphragm 15 Gy
BMT	6				Busulphane 3 × 16 mg/kg/day Cyclophosphamide 4 × 0.2 g/kg/day	
CML AML ALL	2 2 2					TBI 12 Gy (1 pt)
STS II/III	3				CWS 90 Ifosfamide 37.5 g/m^2^ (Actinomycin D, vincristine) Dacarbazine 2.25 g/m^2^ Cyclophosphamide 5 g/m^2^	Supradiaphagm 20 Gy (1 pt) Infradiaphragm (49.6 Gy—1 pt, 25 Gy—1 pt)
NHL III	1				LMB Cyclophosphamide 5.8 g/m^2^	Infradiaphragm 15 Gy
Wilms tumor	6				SIOP (Actinomycin D, vincristine, and epirubicine)	Infradiaphagm 15 Gy—2 pts 20 Gy—2 pts 25 Gy—1 pt 40 Gy—1 pt
III-5 V-1					Ifosfamide 36, actinomycin D, and vincristine	

MR	12	8.04 ± 5.5	18.36 ± 5.7	8.7 ± 4.4		
AML	2				AML—BFM 90 Cytarabine 44.7 g/m^2^	CNS—18 Gy
STS II	3				CWS 96 Ifosfamide 37.5 g/m^2^—1 pt 42 g/m^2^—1 pt 48 g/m^2^—1 pt	Supradiaphragm 20 Gy
NBL II	2				PACE Cisplatin—2 g/m^2^	—
NHL II	5				BFM 95 Cyclophosphamide 3 g/m^2^ Ifosfamide 8 g/m^2^	—

LR	33	7.8 ± 5.3	17.25 ± 5.0	9.0 ± 4.8		
ALL	22				BFM 90 Cyclophosphamide—3.0 g/m^2^	CNS 12 Gy—5 pts CNS 18 Gy—2 pts
Germinal tumors	7				Without cht—4 TGM 95 (Cisplatin, ifosfamide, and etoposide)	Unilateral ovariectomy (7)
Wilms tumor II	5				Actinomycin D, vincristine	

HR: high risk group, MR: medium risk group, LR: low risk group, HL: Hodgkin lymphoma, BMT: bone marrow transplantation, CML: chronic myeloid leukemia, AML: acute myeloid leukemia, ALL: acute lymphoblastic leukemia, STS: soft tissue sarcoma, NHL: non-Hodgkin lymphoma, NBL: neuroblastoma, CNS: central nervous system, and TBI: total body irradiation.

**Table 2:** Serum levels of FSH, LH, E2, and AMH in female cancer survivors according to risk groups and comparison to control group.

Study group	*n*	Hormones	*X*	*M*	SD	*P*
Whole group	83	FSH (mIU/mL)	12.24	7.01	19.41	0.001
HR	38		18.11	7.64	28.71	0.005
MR	12		7.43	7.16	2.77	0.964
LR	33		8.95	6.44	9.63	0.679
Control	34		5.36	5.15	1.89	

Whole group	83	LH (mIU/mL)	7.73	4.49	11.32	0.449
HR	38		10.23	4.10	17.01	0.106
MR	12		5.34	4.42	4.33	1
LR	33		6.44	4.77	5.15	0.924
Control	34		5.52	4.68	4.1	

Whole group	83	E2 (pg/mL)	45.42	32.5	54.72	0.057
HR	38		52.21	33.29	69.20	1
MR	12		51.39	17.60	75.46	0.999
LR	33		38.38	33.20	28.99	0.491
Control	34		53.32	47.39	41.14	

Whole group	83	AMH (pmol/L)	16.7	11.58	14.13	0.001
HR	38		14.14	9.89	13.26	0.001
MR	12		14.82	8.54	16.19	0.019
LR	33		19.44	19.00	13.96	0.053
Control	34		27.03	25.26	12.31	

HR: high risk, MR: medium risk, LR: low risk, FSH: follicle-stimulating hormone, LH: luteinizing hormone, E2: estradiol, AMH: anti-Müllerian hormone, *X*: average value, and *M*: median.

**Table 3:** Hormone levels in female cancer survivors classified to the high risk group.

Analyzed HR group	*n*	Hormones	*X*	*M*	SD	*P*
HL rtx −	12	FSH (mIU/mL)	7.07	7.0	2.78	0.647
HL rtx +	10		8.53	6.9	3.25	0.045
BMT	6		42.55	42.55	26.55	0.034
Solid tumors	9		10.55	7.9	14.68	0.068
Control	34		5.35	5.15	1.89	

HL rtx −		LH (mIU/mL)	6.13	3.85	6.67	0.283
HL rtx +			3.63	3.00	2.05	0.483
BMT			26.31	15.20	28.23	0.001
Solid tumors			6.45	7.0	5.93	0.494
Control			5.52	4.68	4.1	

HL rtx −		E2 (pg/mL)	69.72	42.79	101.82	0.628
HL rtx +			47.34	33.09	32.24	0.578
BMT			26.25	22.74	17.26	0.021
Solid tumors			40.91	17.6	47.29	0.670
Control			53.32	47.39	41.14	

HL rtx −		AMH (pmol/L)	23.17	25.10	15.25	0.042
HL rtx +			14.15	11.90	8.10	0.037
BMT			3.37	2.62	2.32	0.001
Solid tumors			19.21	8.14	16.30	0.05
Control			27.03	25.26	12.31	

HR: high risk, HL: Hodgkin lymphoma, rtx: radiotherapy, BMT: bone marrow transplantation, FSH: follicle-stimulating hormone, LH: luteinizing hormone, E2: estradiol, AMH: anti-Müllerian hormone, *X*: average value, and *M*: median.
